# GDNF content and NMJ morphology are altered in recruited muscles following high‐speed and resistance wheel training

**DOI:** 10.1002/phy2.235

**Published:** 2014-02-25

**Authors:** Amy Morrison Gyorkos, John M. Spitsbergen

**Affiliations:** 1 Department of Biological Sciences, Western Michigan University, 1903 W Michigan Ave., Kalamazoo, 49008‐5410, Michigan

**Keywords:** Fast‐twitch, GDNF, NMJ, skeletal muscle, voluntary exercise

## Abstract

Glial cell line‐derived neurotrophic factor (GDNF) may play a role in delaying the onset of aging and help compress morbidity by preventing motor unit degeneration. Exercise has been shown to alter GDNF expression differently in slow‐ and fast‐twitch myofibers. The aim was to examine the effects of different intensities (10, 20, ~30, and ~40 m·min^−1^) of wheel running on GDNF expression and neuromuscular junction (NMJ) plasticity in slow‐ and fast‐twitch myofibers. Male Sprague‐Dawley Rats (4 weeks old) were divided into two sedentary control groups (CON4 week, *n* = 5 and CON6 week, *n* = 5), two involuntary running groups, one at a low velocity; 10 m/min (INVOL‐low, *n* = 5), and one at a higher velocity; 20 m/min (INVOL‐high, *n* = 5), and two voluntary running groups with resistance (VOL‐R, *n* = 5, 120 g), and without resistance (VOL‐NR,* n* = 5, 4.5 g). GDNF protein content, determined by enzyme‐linked immunosorbent assay (ELISA), increased significantly in the recruited muscles. Plantaris (PLA) GDNF protein content increased 174% (*P *<**0.05) and 161% (*P *<**0.05) and end plate‐stained area increased 123% (*P *<**0.05) and 72% (*P *<**0.05) following VOL‐R, and VOL‐NR training, respectively, when compared to age‐matched controls. A relationship exists between GDNF protein content and end plate area (*r* = 0.880, *P* < 0.01, *n* = 15). VOL‐R training also resulted in more dispersed synapses in the PLA muscle when compared to age‐matched controls (*P *<**0.05). Higher intensity exercise (>30 m/min) can increase GDNF protein content in fast‐twitch myofibers as well as induce changes in the NMJ morphology. These findings help to inform exercise prescription to preserve the integrity of the neuromuscular system through aging and disease.

## Abstract (precis)

Higher intensity exercise (>30 m/min) can increase glial cell line‐derived neurotrophic factor (GDNF) protein content in fast‐twitch myofibers as well as induce changes in the neuromuscular junction (NMJ) morphology. These findings help to inform exercise prescription to preserve the integrity of the neuromuscular system through aging and disease.

## Introduction

The term sarcopenia was first coined in 1989 by Rosenberg to describe the age‐related decline in skeletal muscle mass (Rosenberg 1997). It has since been shown that muscle mass alone may be a weak predictor of disability and mortality (Visser et al. 2005; Goodpaster et al. 2006; Newman et al. 2006). A relationship exists between the loss of muscle mass and loss of strength, but it is not linear due to a faster progression of the latter, leading to diminished muscle quality (muscle strength per unit of muscle mass) (Goodpaster et al. 2006; Delmonico et al. 2009). It has been suggested that muscle quality is a much better independent predictor of functional decline and mortality than loss of muscle mass alone and attention to this factor may be vital to delaying morbidity in the aging adult (Visser et al. 2005; Goodpaster et al. 2006; Newman et al. 2006).

One potential underlying cause of diminished muscle quality is the plasticity of the aging neuromuscular system, as seen with changes in motor units (MUs), peripheral nerves, and the neuromuscular junction (NMJ). As with loss of muscle mass and strength, a reduction of 1% of total MUs per year can be seen beginning as early as the third decade of life and this loss increases exponentially through the sixth decade (Tomlinson and Irving 1977). It has been predicted that between the age of 20–90, an 80% loss of MUs occurs with an accelerated rate of loss after the age of 60 (Wang et al. 1999). Many studies on human and animal aging have shown a preferential loss of the largest and fastest conducting MUs (Hashizume et al. 1988; Knox et al. 1989; Ansved and Larsson 1990; Wang et al. 1999) causing atrophy of type II myofibers more so than type I (Fiatarone et al. 1990; Klitgaard et al. 1990; Larsson et al. 1991). While the mean cross‐sectional area (CSA) of type I myofibers exceeds that of type II by 20% in the third and fourth decades of life (Brooke and Engle 1969), by the eighth decade of life, type II CSA is less than 50% of that of type I (Tomonaga 1977).

As a compensatory mechanism to maintain force production, collateral sprouting of nearby surviving motor axons reinnervate some, but not all type II myofibers (Pestronk et al. 1980). This causes the surviving MUs to increase in size leading to declines in force steadiness and fine motor control (Tracy et al. 2004), declines in motor conduction velocities in peripheral nerve (Wang et al. 1999), and a decline in MU firing rate at effort levels relevant to activities of daily living (Ling et al. 2009).

Physical activity, and specifically resistance training, has been found to be a reliable treatment to slow or reverse the declines observed in skeletal muscle through the aging process (Peterson et al. 2010). Older sedentary individuals have been found to be twice as likely to develop severe sarcopenia compared to age‐matched active individuals (Janssen et al. 2002). Resistance training has been found to significantly increase muscle strength, mass, and functional mobility equally in both men and women (Leenders et al. 2013) and even into the ninth decade of life (Fiatarone et al. 1990). In addition, resistance training has the ability to upregulate protein synthesis as much as 50% in mixed myofibers in response to progressive overload (Yarasheski et al. 1999) and to induce type II myofiber hypertrophy by initiating satellite cell proliferation, differentiation, and fusion of new myonuclei into existing myofibers (Leenders et al. 2013). Furthermore, only resistance training versus a low intensity run‐ and swim‐training had the ability to maintain the mean fiber area of large type II b fibers similar to young controls (Klitgaard et al. 1990).

One possible mechanism underlying the positive adaptations observed following resistance training in type II myofibers is the activity‐induced protection of large MUs. Denervation of the motor neuron has been shown to precede, and to be a necessary prerequisite for, atrophy of the innervated myofiber (Deschenes et al. 2010). In addition, it has been postulated that the net loss of myofibers in sarcopenia may be due to an impaired capacity for axonal reinnervation of denervated myofibers (Aagaard et al. 2010). Therefore, if higher intensity exercise can recruit type II myofibers, perhaps the activity‐induced protection of large MUs can prevent atrophy, and loss of type II myofibers, slowing or delaying the aging process.

A likely candidate to promote the survival of MUs following exercise is a neurotrophic factor named glial cell line‐derived neurotrophic factor (GDNF). GDNF is produced in target tissues of neurons, including skeletal muscle (Suzuki et al. 1998), and acts in a retrograde fashion to exert nourishing effects (Yan et al. 1995). GDNF has been shown to be the most potent neurotrophic factor in promoting the survival of motor neurons and is the only candidate known to date to prevent motor neuron atrophy (Lin et al. 1993; Henderson et al. 1994). It has also been shown to regulate presynaptic and postsynaptic plasticity with a number of effects pertinent to sarcopenia, including causing hyperinnervation by inducing terminal sprouting, providing continuous synaptic remodeling, increasing end plate complexity and size, and protecting large MUs from degeneration (Andonian and Fahim 1987; Lin et al. 1993; Mohajeri et al. 1999; Keller Peck et al. 2001; Zwick et al. 2001).

GDNF expression has been shown to increase following 2 weeks of training in an activity‐dependent manner, but only in presumably recruited muscles (Wehrwein et al. 2002; McCullough et al. 2011). Low intensity walk training was able to increase GDNF in slow‐twitch myofibers, but decreased GDNF in fast‐twitch myofibers (McCullough et al. 2011). In addition, GDNF has been reported to increase following higher intensity exercise in fast‐twitch muscle fibers when comparing swim verse run training (Gyorkos 2014). The difference in GDNF protein content, however, might have been due to the difference between modes of exercise rather than to intensity changes. In order to clarify that the intensity is the factor regulating GDNF in fast‐twitch skeletal muscle, different intensities of exercise with the same mode of training must be compared. Therefore, it is our hypothesis that a higher intensity exercise, such as voluntary wheel training, will recruit fast‐twitch myofibers and subsequently alter GDNF protein content as well as induce plasticity of the NMJ.

## Methods

### Animals

All animal experiments were performed in accordance with the “Guide for the Care and Use of Laboratory Animals” (National Research Council) and protocols were approved by the Institutional Animal Care, and Use Committee at Western Michigan University. Four‐week old male Sprague‐Dawley rats (*n* = 30) were housed with 12:12‐h light‐dark. Rats were randomly divided into two sedentary control groups (CON4 week, *n* = 5 and CON6 week, *n* = 5), two involuntary running groups, one at a low velocity; 10 m/min (INVOL‐low, *n* = 5) and one at a higher velocity; 20 m/min (INVOL‐ high, *n* = 5), and two voluntary running groups, one with resistance (VOL‐R, *n* = 5, 120 g), and one without resistance (VOL‐NR, *n* = 5, 4.5 g) (Table 1).

**Table 1.** Study design summary

	Number	Speed (m·min^−1^)	Duration (km·day^−1^)	Length of time (week)
CON4	5	–	–	2
CON6	5	–	–	2
INVOL‐low	5	10	1.2	2
INVOL‐high	5	20	1.5	2
VOL‐R	5	30	1.5	2
VOL‐NR	5	40	2.2	2

Summary of study design for sedentary and exercised rats. The CON4 week and CON6 week groups were housed individually and remained sedentary for 2 weeks. The exercise groups were also housed individually, but were exercised daily for 2 weeks on a voluntary (VOL‐) or involuntary (INVOL‐) basis.

### Voluntary training protocol

All rats were housed in a clear polycarbonate living chamber (19″ × 10.5″ × 8″). The chambers in the VOL‐R and VOL‐NR groups were attached to an activity wheel system (Lafayette Instrument, Lafayette, IN) that was freely accessible at all times throughout the study. Voluntary exercise was chosen as the mode of training because rats are internally motivated to run at higher intensities spontaneously, and do not need noxious stimuli or external motivators such as food to induce running (Sherwin 1998; Legerlotz et al. 2008). It also allows the rats to run at night when they are naturally active.

The activity wheels were 35 inches in diameter and attached to a braking system and a counter with an optical sensor that sent distance and velocity information to be stored on a computer. Running distance was calculated by multiplying the number of rotations of the activity wheel by the circumference of the wheel (1.1 m). The wheel activity was continuously recorded for the duration of the study.

No resistance was applied to the VOL‐NR, but a load of 4.5 g was necessary to overcome the inertia of the wheel. Resistance was applied to the VOL‐R and calibrated daily to ensure 120 g of load was added to each wheel continuously for the length of the study. Calibration was accomplished by hanging 120 g of known weight on the wheel bar furthest from the axis and precisely tuning the braking system until the wheel could not be displaced.

### Involuntary training protocol

The involuntary running groups consisted of a low‐velocity running group (INVOL‐low; 10 m·day^−1^, *n* = 5) and a higher velocity running group (INVOL‐high; 20 m·day^−1^, *n* = 5) both being forced to exercise in motorized wheels. The INVOL‐low group ran for five 24 min bouts separated by 5 min rest times as previously published (McCullough et al. 2011; Gyorkos 2014), while the INVOL‐high group ran for five 15 min bouts separated by 5 min rest periods.

### Tissue collection and processing

At the completion of the 2 weeks of training, the rats were weighed and euthanized by CO_2,_ asphyxiation, and thoracotomy. Immediately, the soleus (SOL), plantaris (PLA), and extensor digitorum longus (EDL) were excised from the hindlimbs and weighed. These muscles were chosen in this study to determine any potential differences between predominately slow‐ (SOL) and fast‐twitch (EDL and PLA) myofiber phenotypes (Ariano et al. 1973; Armstrong and Phelps 1984) following exercise.

The muscles on the left side of the body were frozen at normal length in isopentane on dry ice and stored at −80°C, and used for immunohistochemistry. The muscles on the right side of the body were further processed and used for detection of GDNF protein content via enzyme‐linked immunosorbent assay (ELISA), as previously published (McCullough et al. 2011). Each muscle was dipped in liquid nitrogen, smashed into fine powder, and homogenized with sample processing buffer (0.55 mol/L NaCl, 0.02 mol/L NaH_2_PO_4_, 0.08 mol/L Na_2_HPO_4_, 2 mmol/L ethylenediaminetetraacetic acid, 0.1 mmol/L benzethonium chloride, 2 mmol/L benzamidine, 20 KIU/mL aprotinin, 0.5% bovine serum albumin (BSA), and 0.05% Tween‐20). Homogenates were centrifuged and supernatants were collected, and stored at −80°C.

### Immunohistochemistry

The midbelly portions of the muscles were embedded in optimum cutting temperature compound (Sakura Finetek, Torrance, CA) and sectioned using a Leica microtome‐cryostat. Sections were cut at 60 *μ*m for visualization of GDNF localization and NMJ structure, and cut at 10 *μ*m for fiber CSA quantification.

All sections were thaw mounted on HistoBond Microscope Slides (VWR; 195 International, Bridgeport, NJ), vacuum sealed, and stored overnight at 4°C.

### Analysis of GDNF localization and NMJ structure

Rat muscle serial longitudinal sections (60 *μ*m) were fixed in 4% paraformaldehyde at room temperature for 1 h, washed in phosphate buffered saline (PBS) (3 × 5 min), and incubated in buffer containing 10% donkey serum, 4% BSA, 0.5% Triton X‐100 in PBS for 30 min in a humidified chamber at room temperature. Slides were then incubated with a primary polyclonal rabbit *α*‐GDNF (Santa Cruz Biotechnologies; 1:200; Dallas, TX) overnight at 4°C. Slides were washed in PBS the next day and incubated for 2 h at room temperature in a secondary antibody cocktail including donkey *α*‐rabbit conjugated to Alexa Fluor 647 (Molecular Probes, 1:1000; Eugene, OR) to visualize GDNF and *α*‐bungarotoxin directly conjugated to AlexaFluor 488 (Molecular Probes, 1:1000) for end plate visualization. All antibodies were diluted in PBS containing 1% bovine serum albumin and 0.1% triton X‐100. Slides were washed the next day in PBS and mounted in PBS: Glycerol (1:1) for microscopy. Images were viewed with a Zeiss Axiovert 100M confocal microscope (Zeiss LSM 510, Jena, Germany).

The sections stained with *α*‐bungarotoxin were used for quantifying end plate measurements. Fifty random end plates were captured for each muscle (SOL, PLA, and EDL) in the study using the confocal microscope with a C‐Apochromat 63×/1.2 water correction objective. Each end plate was visually analyzed to make certain that it was within the longitudinal border of the myofiber before being scanned and stored. Each captured end plate was analyzed using Image J software as previously described (Deschenes et al. 2006). Briefly, a box was drawn around each end plate with the lines of the box touching each side of the stained area. The area within the box represented the total area and total perimeter measurements of the end plate. This area included the stained as well as the nonstained area interspersed between the end plate clusters. The image was then thresholded to identify the *α*‐bungarotoxin staining and represented the stained area and stained perimeter. Dispersion was measured by dividing the stained area by the total area of the end plate; therefore, a lower percentage would indicate a more dispersed synapse.

### Determination: myofiber CSA

The myofiber CSA of the muscle fibers was determined as previously published (Legerlotz et al. 2008). Using widefield fluorescence microscopy, 125–150 random muscle fibers were captured for each muscle (SOL, PLA, and EDL) in this study. Each muscle fiber was encircled and analyzed using Image J software (http://rsbweb.nih.gov/ij/).

### Statistics

All statistical analyses were performed using SPSS statistical software (http://www-01.ibm.com/software/analytics/spss/). Descriptive statistics were calculated to define means and standard errors for all variables. The results were reported as the mean ± standard error of the mean (SEM). Data were analyzed using a one‐way analysis of variance (ANOVA) and Tukey's post hoc comparison to test for statistical significant differences between groups. Linear regression analysis was performed on the individual samples to evaluate the association between variables. Differences were considered statistically significant at *P* < 0.05.

## Results

### Training

On a daily average, the VOL‐NR group ran ~33% faster (~40 m·min^−1^ vs. ~30 m·min^−1^; *P* < 0.05) and ~46% longer (~2.2 km·day^−1^ vs. ~1.5 km·day^−1^; *P* < 0.05) than the VOL‐R group. The amount of work averaged over the 2 weeks of training was similar between groups when factoring in the 120 g of resistance added to the VOL‐R group using a previously published equation: *W* = force (N) × distance (msec)/body wt (kg) (Ishihara et al. 1998). The involuntary running protocols matched closely in running distance for INVOL‐low (10 m·min^−1^) and INVOL‐high groups (20 m·min^−1)^ as they ran for 1.2 km·day^−1^ and 1.5 km·day^−1^, respectively.

### Animal body weight and muscle weights

Body weight decreased (*P *<**0.05) following all of the exercise training protocols with the exception of VOL‐R training (Table 2). Although, the absolute tissue weight was significantly altered in all three muscle groups (SOL, PLA, EDL) depending on the exercise, only the relative weight of the PLA muscle was increased (*P *<**0.05) following voluntary exercise (VOL‐R and VOL‐NR). The relative weight of the SOL and EDL muscles remained unaltered following exercise protocols when compared to sedentary controls.

**Table 2.** Animal body and muscle weights (Values are means ±SEM)

	Body wt (g)	Tissue wt (mg)	Relative muscle wt (mg/g)
SOL			
Control	264 ± 12	149 ± 19	0.56 ± 0.04
INVOL‐low	217 ± 14*	113 ± 32*	0.52 ± 0.05
INVOL‐high	205 ± 15*	95 ± 28*	0.46 ± 0.07
VOL‐R	272 ± 14	133 ± 23	0.49 ± 0.05
VOL‐NR	230 ± 15*	128 ± 20*	0.55 ± 0.06
PLA			
Control	264 ± 12	234 ± 21	0.88 ± 0.03
INVOL‐low	217 ± 14*	258 ± 19	1.10 ± 0.04
INVOL‐high	205 ± 15*	240 ± 28	1.17 ± 0.06
VOL‐R	272 ± 14	351 ± 18*	1.29 ± 0.03*
VOL‐NR	230 ± 15*	293 ± 26*	1.27 ± 0.06*
EDL			
Control	264 ± 12	142 ± 18	0.54 ± 0.06
INVOL‐low	217 ± 14*	119 ± 16*	0.57 ± 0.07
INVOL‐high	205 ± 15*	125 ± 18*	0.57 ± 0.05
VOL‐R	272 ± 14	158 ± 19	0.58 ± 0.04
VOL‐NR	230 ± 15*	131 ± 26	0.57 ± 0.06

Rat body weight (g), absolute (mg), and relative tissue weights (mg/g) were measured in sedentary control animals and following training protocols. The relative muscle weight of the PLA increased following voluntary exercise (VOL‐R and VOL‐NR). Symbols denote statistical significance (*P* ≤ 0.05) compared to control (*).

### Effects of exercise on PLA muscle

#### GDNF protein content in PLA

**Figure 1.**
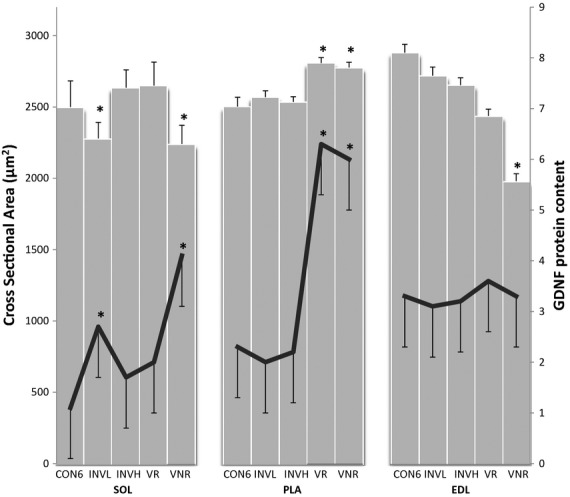
GDNF protein content increases in recruited muscles. Quantification of GDNF was accomplished via ELISA. Cross‐sectional area (CSA; bar graph) was measured in 125–150 random SOL, EDL, and PLA fibers that were captured by widefield microscopy and analyzed using ImageJ software. GDNF protein content (pg/mg per tissue weight; line graph), determined by ELISA, was significantly increased in all recruited fibers of the SOL and PLA muscles, evidence by CSA decreasing and increasing, respectively. Increases in GDNF protein content were observed in SOL muscle following INVOL‐low and VOL‐NR training protocols. Increases in GDNF were observed in PLA muscle following both voluntary training. The EDL did not appear to be recruited following any training and no changes in GDNF were observed. Values are displayed as the mean ± SEM. Asterisk (*) indicates a significant (*P* ≤ 0.05) difference from the age‐matched control group. Key: CON6 (CON6 week), INVL (INVOL‐low), INVH (INVOL‐high), VR (VOL‐R), and VNR (VOL‐NR).

CSA was measured as an indicator of muscle recruitment where slow‐ and fast‐twitch myofibers have been shown to respond differently by decreasing and increasing CSA, respectively (Waerhaug et al. 1992; Deschenes et al. 1993, 2010). The CSA of the PLA myofibers increased significantly following both VOL‐R and VOL‐NR exercise when compared to age‐matched sedentary controls (Fig. 1; bar graph). In those recruited muscles, GDNF protein content increased 174% (*P *<**0.05) following VOL‐R exercise and 161% (*P *<**0.05) following VOL‐NR exercise when compared to age‐matched sedentary controls (Fig. 1; line graph). There were no significant differences in PLA GDNF protein content or CSA between VOL‐R and VOL‐NR training groups, or following involuntary exercise compared to age‐matched sedentary controls.

#### End plate measurements in PLA

**Figure 2.**
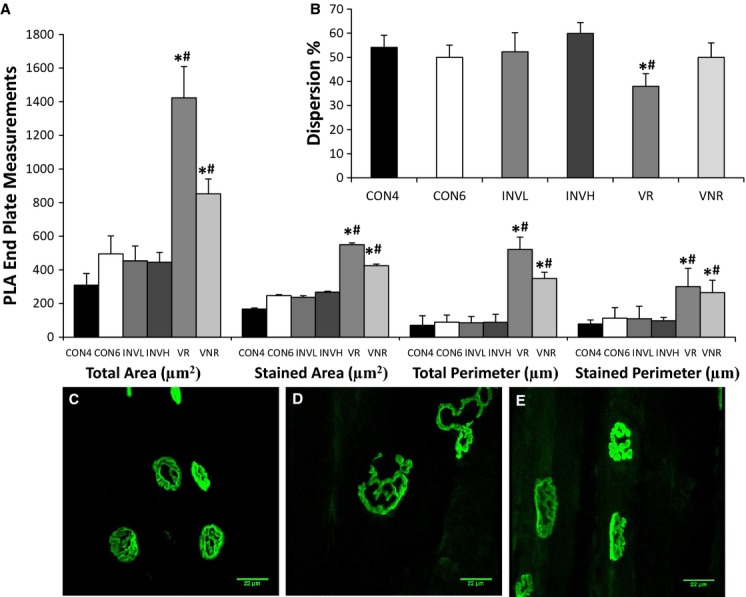
Effects of exercise on postsynaptic end plate morphology in PLA muscle. Cross sections (60 *μ*m) of SOL muscle fibers were stained with *α*‐bungarotoxin for quantification and visualization. Total and stained area (*μ*m^2^), and total and stained perimeter (*μ*m) were altered following VOL‐R and VOL‐NR training when compared to age‐matched sedentary controls (A). In addition, VOL‐R training resulted in more dispersed synapses compared to the 6 week control group (B). End plates are shown for the 6 week‐CON (C), VOL‐R (D), and VOL‐NR (E) groups. Note the dispersion observed following VOL‐R training (D). Values are displayed as the mean ± SEM. Asterisk (*) indicates a significant (*P* ≤ 0.05) difference from the age‐matched sedentary control group. Number symbol (^#^) indicates a significant (*P* ≤ 0.05) difference from the 4 week‐control group. Key: CON4 (CON4 week), CON6 (CON6 week), INVL (INVOL‐low), INVH (INVOL‐high), VR (VOL‐R), and VNR (VOL‐NR).

Immunohistochemistry revealed that all of the measurements quantified for end plates in the PLA myofibers were altered following voluntary exercise. Total area, stained area, total perimeter, and stained perimeter were all increased in the VOL‐R and VOL‐NR groups compared to age‐matched controls (*P *<**0.05; Fig. 2). The end plate stained area increased 123% (*P *<**0.05) and 72% (*P *<**0.05) following VOL‐R and VOL‐NR training, respectively, when compared to age‐matched controls. Voluntary training groups showed increased end plate measurements compared to age‐matched controls. The end plates increased even further following VOL‐R training when compared to VOL‐NR training. A positive relationship exists between PLA GDNF protein content and end plate area (*r* = 0.880, *P* < 0.01, *n* = 15) (Figs. 4, 5). VOL‐R training also resulted in more dispersed synapses when compared to VOL‐NR training and age‐matched control groups (*P *<**0.05; Fig. 2 inset). There were no significant differences observed in end plate measurements following involuntary exercise compared to age‐matched sedentary controls.

### Effects of exercise on SOL muscle

#### GDNF protein content in SOL

The CSA of the SOL myofibers decreased following INVOL‐low and VOL‐NR training when compared to age‐matched sedentary controls (Fig. 1; bar graph). SOL GDNF protein content increased 145% (*P *<**0.05) and 272% (*P *<**0.05) following INVOL‐low and VOL‐NR training, respectively, when compared to age‐matched controls (Fig. 1; line graph). There were no significant differences observed in measurements of fiber CSA and GDNF following INVOL‐high and VOL‐R training compared to age‐matched sedentary controls.

#### End plate measurements in SOL

**Figure 3.**
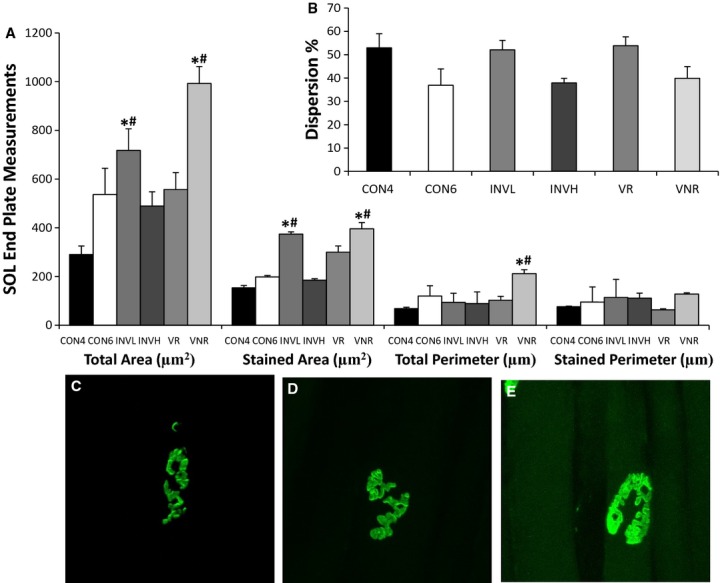
Effects of exercise on postsynaptic end plate morphology in SOL muscle. Cross sections (60 *μ*m) of SOL muscle fibers were stained with *α*‐bungarotoxin for quantification and visualization. Total and stained area (*μ*m^2^) were altered following INVOL‐low and VOL‐NR training when compared to age‐matched sedentary controls (A). In addition, total perimeter was increased following VOL‐NR training. No significant differences in dispersion were observed (B). End plates are shown for the 6 week CON (C), INVOL‐low (D), and VOL‐NR (E) groups. Values are displayed as the mean ± SEM. Asterisk (*) indicates a significant (*P* ≤ 0.05) difference from the age‐matched sedentary control group. Number symbol (^#^) indicates a significant (*P* ≤ 0.05) difference from the 4 week control group. Key: CON4 (CON4 week), CON6 (CON6 week), INVL (INVOL‐low), INVH (INVOL‐high), VR (VOL‐R), and VNR (VOL‐NR).

**Figure 4.**
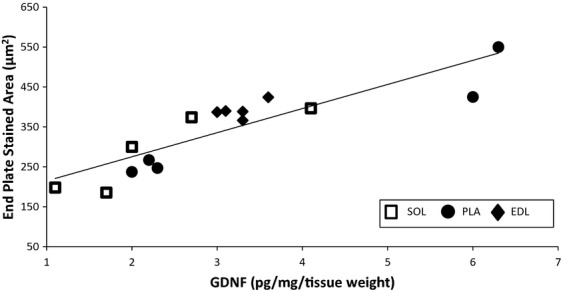
Correlation between GDNF levels in skeletal muscle and end plate‐stained area. A relationship exists between GDNF protein content (pg/mg/tissue weight) and end plate‐stained area (*μ*m^2^) (*r* = 0.880, *P* < 0.01). Each point represents one of the three muscles (SOL, PLA, EDL) in one of five groups (CON6 week, INVOL‐low, INVOL‐high, VOL‐R, and VOL‐NR), giving 15 data points (*n* = 15).

**Figure 5.**
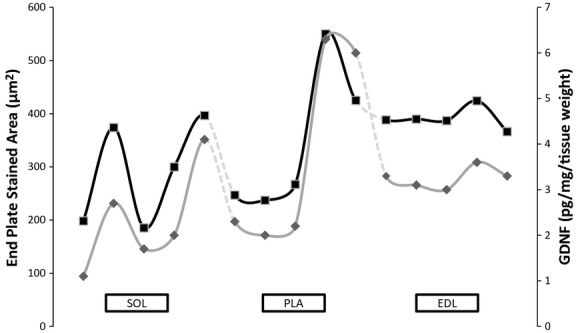
Relationship between GDNF protein content and end plate‐stained area. This graph displays the close relationship between GDNF and end plate area in the SOL, PLA, and EDL muscles.

The total and stained area and total perimeter of the SOL end plates increased following INVOL‐low and VOL‐NR training (Fig. 3). Stained area of SOL end plates increased 89% (*P *<**0.05) and 100% (*P *<**0.05) following INVOL‐low and VOL‐NR training, respectively, when compared to age‐matched sedentary controls. There were no significant differences in dispersion of the SOL end plates. A relationship exists between GDNF protein content and end plate area (*r* = 0.880, *P* < 0.01, *n* = 15) (Figs. 4, 5). There were no significant differences observed in end plate measurements following INVOL‐high and VOL‐R training compared to age‐matched sedentary controls.

### Effects of exercise on EDL muscle

#### CSA and GDNF protein content in EDL

The CSA of the EDL myofibers decreased following VOL‐NR training compared to age‐matched sedentary controls (Fig. 1; bar graph). There were no other significant differences observed in measurements of fiber CSA and GDNF (Fig. 1; line graph) compared to age‐matched sedentary controls.

#### End plate measurements in EDL

There were no significant differences observed among end plate measurements when compared to age‐matched sedentary controls. There were no differences in the dispersion of the EDL end plates when compared to controls. A relationship exists between GDNF protein content and end plate area (*r* = 0.880, *P* < 0.01, *n* = 15) (Figs. 4, 5).

## Discussion

This is the first study to our knowledge that has shown an increase in GDNF protein content in fast‐twitch myofibers. GDNF levels in fast‐twitch myofibers have been found to significantly decrease following short‐term exercise at low velocities (10 m·day^−1^) (McCullough et al. 2011). We were able to show that by increasing both the velocity (30–40 m·day^−1^) and the resistance (120 g) of the training, the fast‐twitch myofibers of the PLA muscle were recruited, and subsequently increased GDNF content.

Rats will spontaneously and naturally run at speeds close to their maximal aerobic capacity in short bouts mimicking sprint training (Shepherd and Gollnick 1976; Rodnick et al. 1989). It has been shown that the high‐intensity nature of voluntary exercise can induce increases in enzymatic activity in fast‐twitch myofibers and that these changes are unlikely caused by hormonal changes, but may be a result of different patterns of muscle recruitment following higher intensity training (Bagby et al. 1986; Rodnick et al. 1989). Speeds as low as 25–40 m/min have been found to significantly deplete glycogen, increase lactate accumulation and to increase cytochrome *c* concentration in fast‐twitch myofibers (Armstrong et al. 1974; Baldwin et al. 1977; Dudley et al. 1982) indicating recruitment of those myofibers. In this study, rats were able to reach speeds between ~30 and 40 m/min on average through voluntary exercise, which reaches the intensity threshold for recruitment (Armstrong et al. 1974; Baldwin et al. 1977; Dudley et al. 1982).

Addition of a load of 120 g of resistance within the first 2 weeks of this study was enough to induce hypertrophy in the PLA muscle as previously reported (Ishihara et al. 1998; Legerlotz et al. 2008). An increase in the CSA of the PLA muscle has been shown to follow an increase in load (Ishihara et al. 1998; Legerlotz et al. 2008) or an increase in duration (Kariya et al. 2004) of the training period. The voluntary exercise reached an intensity that was high enough to recruit the fast‐twitch myofibers in both training groups and may have contributed to an increase in GDNF protein content in the PLA muscle. This is clinically relevant as this indicates that higher intensity training may be able to support the innervating neurons of those vulnerable fast‐twitch myofibers through increased expression of neurotrophic factors.

We were also able to show that the training altered the end plate morphology of the recruited muscles, consistent with previous findings that exercise can induce changes to the postsynaptic apparatus in rats (Waerhaug et al. 1992; Deschenes et al. 1993; McCullough et al. 2011). Deschenes et al. (1993) observed increases in the dispersion of end plates of fast‐twitch myofibers following higher intensity training. This study showed similar results as the PLA end plates were more dispersed following VOL‐R training, but not following VOL‐NR training. It has been postulated that this pattern of dispersion following higher intensity exercise may be the result of longer and more complex arborization of the presynaptic terminal, compared to the shorter primary branches observed following lower intensity exercise (Deschenes et al. 1993). These results suggest that the intensity of exercise alters the NMJ of slow‐ and fast‐twitch myofibers in different ways.

GDNF, in the absence of exercise, has also been found to cause continuous synaptic remodeling of the NMJ by inducing hyperinnervation, increasing end plate size, and complexity, and maintaining the postsynaptic apparatus (Keller Peck et al. 2001; Zwick et al. 2001; Wang et al. 2002). The results of this study are consistent with those findings as GDNF protein content was positively correlated with end plate size. It has been postulated that presynaptic changes, such as terminal sprouting, increased neurotransmission stores, and quantal storage and release may warrant changes in the postsynaptic apparatus in order to enhance communication and efficiency at the NMJ (Stephens and Taylor 1972; Dorlöchter et al. 1991; Hill et al. 1991; Keller Peck et al. 2001; Zwick et al. 2001).

It is noteworthy that the muscles that were not actively recruited; PLA during involuntary training, SOL during INVOL‐high, and VOL‐R training, and EDL following all training, with the exception of VOL‐NR, displayed no changes in NMJ morphology, and GDNF levels remained similar to those in sedentary controls. These findings provide further support that the regulation of GDNF is activity‐dependent, as its expression relies on the recruitment of the myofibers during physical activity (Wehrwein et al. 2002; McCullough et al. 2011).

Physical activity levels and intensity decline with aging, with an associated decline in skeletal muscle mass and strength (Rosenberg (1997; Janssen et al. 2002). Those losses in mass and strength result in significant public health problems with associated increases in falls, fractures, and frailty, declines in functional mobility, and independence, leading to a diminished quality of life, morbidity, and mortality (Aniansson et al. 1984; Nevitt et al. 1989; Roubenoff 2001; Janssen et al. 2002). It has been postulated that there is no other decline in aging that is as dramatic or as significant as that of lean body mass (Rosenberg 1997). The decline of 3–8% of muscle mass per decade begins as early as 30 years of age with ~45% of the U.S. population sarcopenic and 20% functionally disabled as they approach 65 years of age (Manton and Gu 2001). The population over 60 years old is predicted to triple in the next 50 years and with it comes diminished quality of life and disability, leading to economic costs for government reimbursed health care, unless underlying mechanisms are identified and preventative measures are taken (Janssen et al. 2002).

Being physically active, on the other hand, has been shown to compress morbidity and have a “squaring off” effect on the disability and mortality curves so that people can live longer productive lives, and die after a limited period of disability (Fries 2002). In this context, exercise has been compared to a nonpharmacological fountain of youth (Joyner and Barnes 2013). In addition, physical fitness has been shown to be the number one independent risk factor for both all‐cause and cardiovascular morbidity (Kodama et al. 2009).

GDNF is a muscle‐derived, activity‐dependent factor that may play a critical role in the positive outcomes of exercise. GDNF may act to promote the survival of MUs and, therefore, play a pertinent role in delaying the onset of aging of the neuromuscular system. The effects of exercise as a regulatory factor of GDNF expression deserves continued research in order to inform exercise prescription to induce changes in those myofibers that are most susceptible to degeneration, preserving nerve and muscle function, and protecting the neuromuscular system.

## Conflict of Interest

None declared.
